# Crystal structure of poly[[aqua­(μ_2_-pyrazine-κ^2^*N*:*N*′)(μ_2_-2,3,5,6-tetra­chloro­benzene-1,4-di­car­boxyl­ato-κ^2^*O*^1^:*O*^4^)copper(II)] hemihydrate]

**DOI:** 10.1107/S2056989025003457

**Published:** 2025-04-24

**Authors:** Hitoshi Kumagai, Satoshi Kawata, Nobuhiro Ogihara

**Affiliations:** a Toyota Central R&D Labs., Inc., 41-1 Yokomichi, Nagakute, Aichi 480-1192, Japan; b Department of Chemistry Fukuoka University 8-19-1 Nanakuma Jonan-ku Fukuoka 814-0180 Japan; Tokyo University of Science, Japan

**Keywords:** crystal structure, hydrogen bonding, C—Cl⋯π inter­action, tetra­chloro­terephthalate, pyrazine, copper

## Abstract

The asymmetric unit of the title compound, {[Cu_2_(C_8_Cl_4_O_4_)_2_(C_4_H_4_N_2_)_2_(H_2_O)_2_]·H_2_O}_*n*_ or {[Cu_2_(Cl_4_bdc)_2_(pyz)_2_(H_2_O)_2_]·H_2_O}_*n*_ comprises of a Cu^II^ ion, one tetra­chloro­benzene­dicarboxyl­ate ion (Cl_4_bdc^2−^), one pyrazine ligand (pyz), and one and a half water mol­ecules. The Cu^II^ ion exhibits a five-coordinated square-pyramidal geometry with a CuN_2_O_3_ coordination environment comprising two oxygen atoms of the Cl_4_bdc^2−^ ligands, one oxygen atom of a water mol­ecule, and two nitro­gen atoms of the pyz ligands. The carboxyl­ate group is almost perpendicular to the benzene ring and shows monodentate coordination to the Cu^II^ ion. The Cu^II^ ions of these units are bridged by both the Cl_4_bdc^2−^ and pyz ligands to form two-dimensional (2D) layers, which are linked by alternating hydrogen-bonding and C—Cl⋯π inter­actions to yield a three-dimensional network.

## Abstract 2

The Cu^II^ ions in this compound form a square-pyramidal coordination environment and are bridged by the Cl_4_bdc^2−^ and pyz ligands to form a two-dimensional (2D) layer. The 2D layers are alternately stacked by hydrogen-bonding and C—Cl⋯π inter­actions to form a three-dimensional network.

## 1. Chemical context

Metal–organic frameworks (MOFs) or coordination polymers (CPs) consist of infinite assemblies of metal ions bridged by organic linkers such as benzendi­carboxyl­ate dianions and are attracting much inter­est due to their applications, for example in gas adsorption, and their optical and magnetic properties (Eddaoudi *et al.*, 2002 ▸; Cheetham *et al.*, 1999 ▸; Lacroix & Nakatani, 1997 ▸; Kitagawa *et al.*, 2004 ▸; Kurmoo, 2009 ▸). We have prepared electrode materials using a benzendi­carboxyl­ate dianion and its analogues (Ogihara *et al.*, 2014 ▸, 2023 ▸; Yasuda & Ogihara, 2014 ▸; Ozawa *et al.*, 2018 ▸; Mikita *et al.*, 2020 ▸), and also magnetic materials that involve polycarboxyl­ates in which the number of carboxyl­ate groups and the distances between them are systematically varied (Kumagai *et al.*, 2001 ▸, 2002 ▸, Kurmoo *et al.*, 2001 ▸, 2003 ▸, 2005 ▸). The selection of metal ions and appropriate bridging ligands is fundamental for the development of a rational synthetic method to prepare these functional materials. We have reported the fine tuning of crystal structures and the properties of these materials, where not only are the metal ions systematically changed, but also the halogen atoms attached to the benzene ring of benzene­dicarboxyl­ate dianions (*R*_4_bdc^2−^; *R* = H, F, Cl, Br) with pyrazine (pyz) or 4,4′-bi­pyridine (bpy) as co-bridging ligands (Kumagai *et al.*, 2012 ▸, 2021 ▸). Among the *M*/Br_4_bdc^2−^/pyz (*M* = Co^II^, Cu^II^, Zn^II^) systems, the Cu^II^ compound showed a different structure and water adsorption/desorption properties due to the different coordination geometry around the Cu^II^ ion (Kumagai *et al.*, 2021 ▸). In this contribution, we focus on the use of the Cl_4_bdc^2−^ dianion and pyz as a co-bridging ligand in the synthesis of a Cu^II^–Cl_4_bdc^2−^ dianion system to observe the structural change that results from the substitution of Br_4_bdc^2−^ for Cl_4_bdc^2−^. Here, we report on the single-crystal structure of [Cu_2_(Cl_4_bdc)_2_(pyz)_2_(H_2_O)_2_](H_2_O).
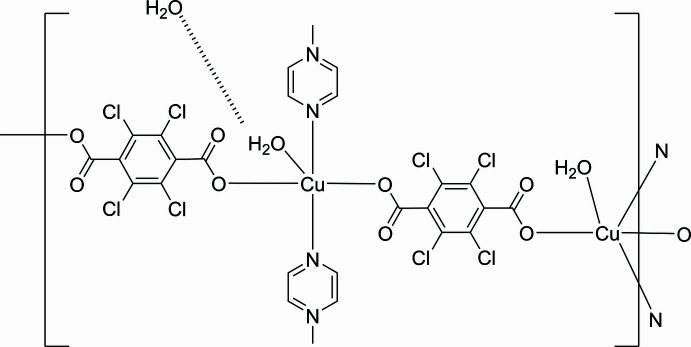


## 2. Structural commentary

**Figure 1:**
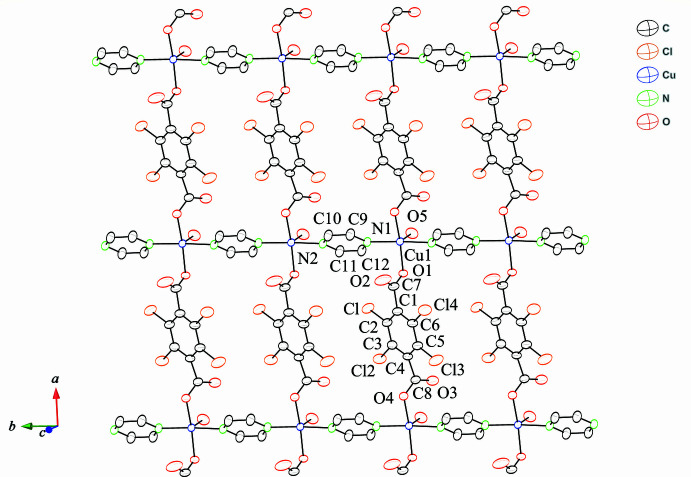
The two-dimensional layered structure of the title compound with the atom-labelling scheme and 50% probability displacement ellipsoids. Hydrogen atoms and the water molecules of crystallization are omitted for clarity.

The asymmetric unit of the title compound, [Cu_2_(Cl_4_bdc)_2_(pyz)_2_(H_2_O)_2_](H_2_O), consists of one Cu^II^ ion, one Cl_4_bdc^2−^ dianion, one pyz ligand, one water molecule coordinated by the metal and ahalf water molecule of crystallization. The key feature of the structure is a two-dimensional (2D) coordination polymer in which CuN_2_O_3_ square pyramids bridged by Cl_4_bdc^2−^ and pyz ligands are arranged in a lyaer, as shown in Fig. 1 ▸. The Cu^II^ ion is penta-coordinated with a square-pyramidal geometry. Pairs of Cl_4_bdc^2−^ and pyz ligands are coord­inated *trans* to each other to give the basal plane of the pyramid, while the coordinated water mol­ecule occupies the apical position. The carboxyl­ate group exhibits monodentate coordination, and the dihedral angle between the benzene ring and the carboxyl­ate group is roughly orthogonal [C6—C1—C7—O1 105.5 (3)°]. We have recently reported a series of 2D layer compounds involving *M*^II^ cations (*M* = Co, Cu, Zn), the tetra­bromo­benzene­dicarboxyl­ate ligand (Br_4_bdc^2−^) and pyz as co-bridging ligands (Kumagai *et al.*, 2021 ▸). While the metal ions exhibit octa­hedral coordination environments in these complexes, the Cu^II^ ion of the title compound has a square-pyramidal geometry. The 2D layers of [*M*(Br_4_bdc)(pyz)(H_2_O)] (*M* = Co, Zn) include pyz mol­ecules between the layers to give [*M*(Br_4_bdc)(pyz)(H_2_O)_2_](pyz) by hydrogen-bonding and π–π stacking inter­actions. On the other hand, the [Cu(Br_4_bdc)(pyz)(H_2_O)_2_] layers contain water mol­ecules due to elongation of the Cu—O(H_2_O) bond, which prevents the π–π stacking inter­actions of the pyz mol­ecules. The Cu—O(H_2_O) bond length of 2.301 (2) Å in the title compound is longer than that of [*M*(Br_4_bdc)(pyz)(H_2_O)_2_] [*M* = Co 2.096 (4) Å, Zn 2.090 (3) Å], but shorter than that in [Cu(Br_4_bdc)(pyz)(H_2_O)_2_] [2.487 (4) Å]. The Cu—N bond lengths of 2.015 (2) and 2.019 (2) Å are similar to that in [Cu(Br_4_bdc)(pyz)(H_2_O)_2_] [2.012 (3) Å]. The Cu⋯Cu separations defined by Cu–Cl_4_bdc^2−^–Cu connectivity and Cu–pyz–Cu connectivity within the chain are 10.98 and 6.78 Å, respectively. While the Cu⋯Cu distance in Cu–pyz–Cu is similar to that in [Cu(Br_4_bdc)(pyz)(H_2_O)_2_] (6.79 Å), the separation for Cu–Cl_4_bdc^2−^–Cu is slightly shorter than that in [Cu(Br_4_bdc)(pyz)(H_2_O)_2_] (11.14 Å). It has been reported that the synthesis of metal complexes using Cl_4_bdc^2−^ gives different structures depending on the synthetic conditions employed (Chen *et al.*, 2011 ▸, 2014 ▸). The title compound was synthesized by the same procedure as that used for the synthesis of the *M*/Br_4_bdc^2−^/pyz (*M* = Co^II^, Cu^II^, Zn^II^) systems; therefore, it is considered that the structural differences are attributable to the halogen atoms attached to the benzene ring. A similar coordination network, [Cu(Cl_4_bdc)(dioxane)(H_2_O)_2_]·(dioxane), with dioxane as co-bridging ligand instead of pyz, has previously been synthesized using different synthetic conditions (He *et al.*, 2009 ▸). The Cu^II^ ion exhibits an octa­hedral coordination environment and an almost rectangular 2D framework with dimensions of *ca*. 11.1 × 7.9 Å defined by the Cu⋯Cu separation. Although the Cu⋯Cu distance for Cu–Cl_4_bdc^2−^–Cu in [Cu(Cl_4_bdc)(dioxane)(H_2_O)_2_] is similar to that for the title compound, the Cu⋯Cu distance for Cu–dioxane–Cu is longer than that for Cu–pyz–Cu due to the large Cu—O(dioxane) bond length of 2.575 (2) Å.

## 3. Supra­molecular features

**Figure 2:**
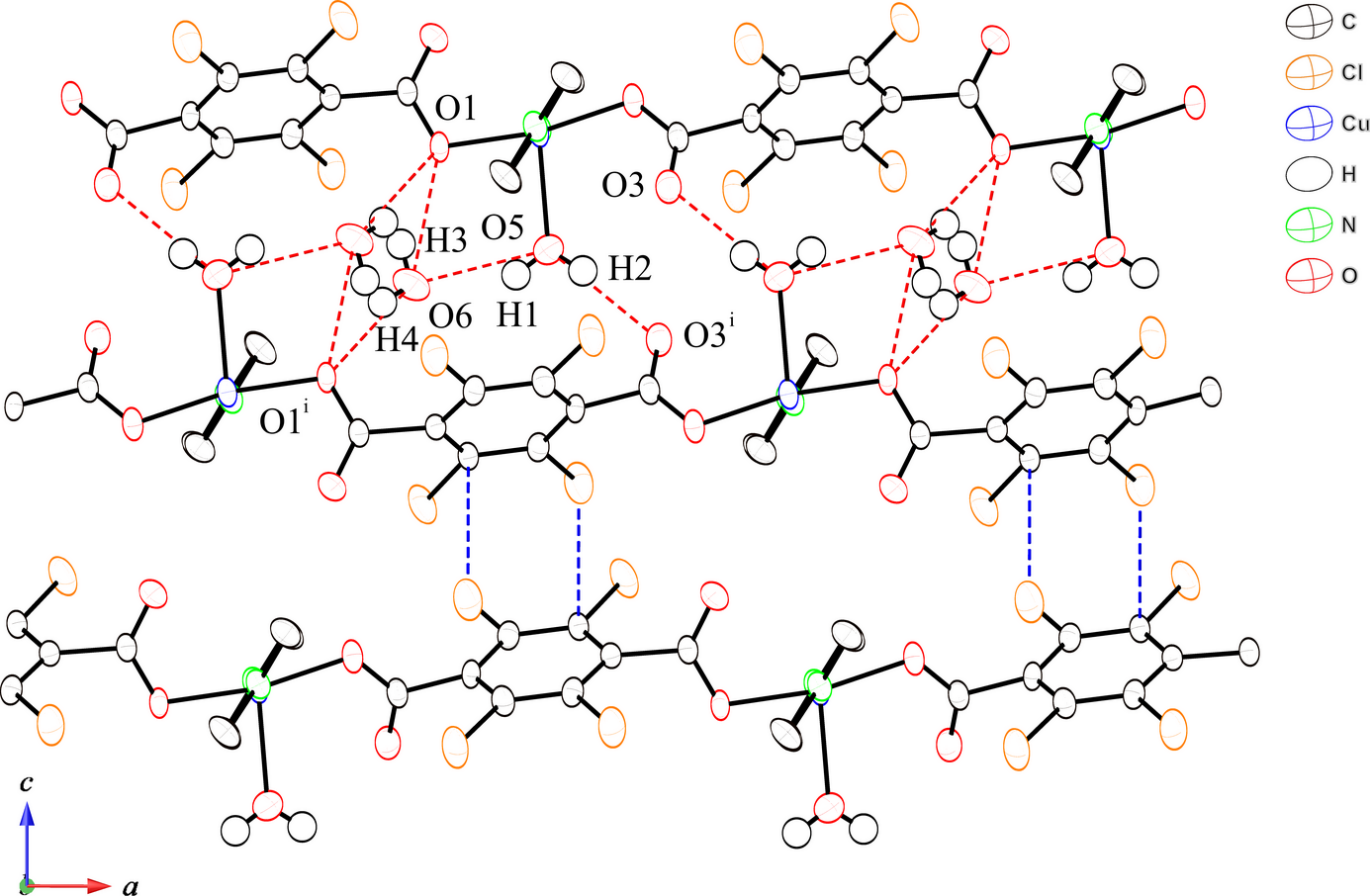
View of inter-mol­ecular hydrogen-bonding and C—Cl⋯π inter­actions along the *b* axis, represented by red and blue dashed lines, respectively.

There are two types of inter­actions between the 2D networks in the crystal structure, namely hydrogen-bonding and other C—Cl⋯π inter­actions. The hydrogen-bonding inter­actions involve water mol­ecules and carboxyl­ate groups. The coord­inated water mol­ecules act as hydrogen-bonding donors and the non-coordinated oxygen atoms of the carboxyl­ate groups of the Cl_4_bdc^2−^ ligands in the adjacent 2D layer act as hydrogen-bonding acceptors (Fig. 2 ▸, Table 1 ▸). The coordinated water mol­ecules also act as hydrogen-bonding donors for the water mol­ecules of crystallization. These molecules, in turn, form hydrogen-bonding interactions with the coordinated oxygen atoms of the Cl_4_bdc^2−^ ligands in the adjacent 2D layers. The other characteristic feature of the structure is C—Cl⋯π inter­actions between the 2D layers. The distances Cl3⋯C6^ii^ [3.370 (3) Å, symmetry code: (ii) −*x* − 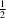, *y* − 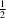, *z* − 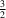] and Cl3⋯centroid of the phenyl ring [3.745 (9) Å] are indicative of C—Cl⋯π inter­actions (Gilday *et al.*, 2015 ▸). The layers are thus alternately stacked by hydrogen-bonding and C—Cl⋯π inter­actions to form a 3D network. The 2D layers in [Cu(Br_4_bdc)(pyz)(H_2_O)_2_] form a 3D network solely by hydrogen-bonding inter­actions *via* water mol­ecules between the 2D layers in which Cu^II^ ions exhibit an octa­hedral coordination geometry. Chemical modification by replacement of the halogen atoms results not only in different coordination geometries of the Cu^II^ ions, but also in different inter-layer inter­actions.

## 4. Database survey

A search of the SciFinder database for structures with Cl_4_bdc^2−^, pyz ligands, and Cu^II^ ions resulted in no complete matches. A search of the Web of Science database for the keywords 2,4,5,6-tetra­chloro-1,3-benzene­dicarb­oxy­lic acid and copper led to two publications that include di­methyl­formamide (FUDPUQ), pyridine (XUWRAJ, FUDPIE) and dioxane (FUDPOK) as co-ligands (He *et al.*, 2009 ▸; Zheng *et al.*, 2009 ▸).

## 5. Synthesis and crystallization

An aqueous solution (5 mL) of copper(II) nitrate trihydrate (0.24 g, 1.0 mmol) was transferred to a glass tube, and an ethanol–water (1:1) mixture (5 mL) of 2,3,5,6-tetra­chloro­benzene­dicarb­oxy­lic acid (0.30 g, 1.0 mmol), NaOH (0.16 g, 2.0 mmol), and pyrazine (0.08 g, 1.0 mmol) was poured into the glass tube without the two solutions being mixed. Blue crystals began to form at ambient temperature in 1 week. One of these crystals was used for X-ray diffraction analysis.

## 6. Refinement

The crystal data, data collection, and structure refinement details are summarized in Table 2 ▸. The hydrogen atoms attached to water mol­ecules were extracted from difference-Fourier maps and refined isotropically. Other hydrogen atoms were located at ideal positions and were refined using a riding model.

## Supplementary Material

Crystal structure: contains datablock(s) I. DOI: 10.1107/S2056989025003457/jp2018sup1.cif

Structure factors: contains datablock(s) I. DOI: 10.1107/S2056989025003457/jp2018Isup2.hkl

Supporting information file. DOI: 10.1107/S2056989025003457/jp2018Isup3.cdx

CCDC reference: 2444566

Additional supporting information:  crystallographic information; 3D view; checkCIF report

## Figures and Tables

**Table 1:** Hydrogen-bond geometry (Å, °)

*D*—H⋯*A*	*D*—H	H⋯*A*	*D*⋯*A*	*D*—H⋯*A*
O5—H1⋯O6	0.79 (2)	2.11 (3)	2.781 (7)	143 (4)
O5—H2⋯O3^i^	0.82 (2)	2.03 (3)	2.827 (3)	163 (5)
O6—H3⋯O1	0.82 (2)	2.04 (5)	2.763 (6)	147 (8)
O6—H4⋯O1^i^	0.82 (2)	2.07 (4)	2.843 (6)	156 (10)

Symmetry code: (i) 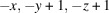.

**Table 2:** Experimental details

Crystal data	
Chemical formula	[Cu_2_(C_8_Cl_4_O_4_)_2_(C_4_H_4_N_2_)_2_(H_2_O)_2_]·H_2_O
*M*_r_	945.07
Crystal system, space group	Monoclinic, *P*2_1_/*n*
Temperature (K)	293
*a*, *b*, *c* (Å)	10.975 (3), 6.7837 (15), 21.803 (4)
β (°)	90.222 (14)
*V* (Å^3^)	1623.3 (6)
*Z*	2
Radiation type	Mo *K*α
μ (mm^−1^)	2.03
Crystal size (mm)	0.20 × 0.20 × 0.20

Data collection	
Diffractometer	Rigaku Mercury70
Absorption correction	Multi-scan (*REQAB*; Rigaku, 2008 ▸)
*T*_min_, *T*_max_	0.429, 0.666
No. of measured, independent and observed [*I* > 2σ(*I*)] reflections	15268, 3684, 3391
*R*_int_	0.030
(sin θ/λ)_max_ (Å^−1^)	0.650

Refinement	
*R*[*F*^2^ > 2σ(*F*^2^)], *wR*(*F*^2^), *S*	0.034, 0.109, 1.18
No. of reflections	3684
No. of parameters	242
No. of restraints	5
H-atom treatment	H atoms treated by a mixture of independent and constrained refinement
Δρ_max_, Δρ_min_ (e Å^−3^)	0.49, −0.67

Computer programs: *CrystalClear* (Rigaku, 2008 ▸), *SIR92* (Altomare *et al.*, 1994 ▸), *SHELXL2018/3* (Sheldrick, 2015 ▸) and *Yadokari-XG 2009* (Wakita, 2009 ▸).

## supplementary crystallographic information

### Poly[[aqua(µ2-pyrazine-κ2N:N')(µ2-2,3,5,6-tetrachlorobenzene-1,4-dicarboxylato-κ2O1:O4)copper(II)] hemihydrate] Crystal data

**Table:**

[Cu_2_(C_8_Cl_4_O_4_)_2_(C_4_H_4_N_2_)_2_(H_2_O)_2_]·H_2_O	*F*(000) = 936
*M**_r_* = 945.07	*D*_x_ = 1.934 Mg m^−^^3^
Monoclinic, *P*2_1_/*n*	Mo *K*α radiation, λ = 0.7107 Å
*a* = 10.975 (3) Å	Cell parameters from 4544 reflections
*b* = 6.7837 (15) Å	θ = 1.9–30.1°
*c* = 21.803 (4) Å	µ = 2.03 mm^−^^1^
β = 90.222 (14)°	*T* = 293 K
*V* = 1623.3 (6) Å^3^	Prism, colorless
*Z* = 2	0.20 × 0.20 × 0.20 mm

### Poly[[aqua(µ2-pyrazine-κ2N:N')(µ2-2,3,5,6-tetrachlorobenzene-1,4-dicarboxylato-κ2O1:O4)copper(II)] hemihydrate] Data collection

**Table:**

Rigaku Mercury70 diffractometer	3391 reflections with *I* > 2σ(*I*)
Detector resolution: 7.314 pixels mm^-1^	*R*_int_ = 0.030
ω scans	θ_max_ = 27.5°, θ_min_ = 1.9°
Absorption correction: multi-scan (*REQAB*; Rigaku, 2008)	*h* = −14→13
*T*_min_ = 0.429, *T*_max_ = 0.666	*k* = −8→8
15268 measured reflections	*l* = −28→28
3684 independent reflections	

### Poly[[aqua(µ2-pyrazine-κ2N:N')(µ2-2,3,5,6-tetrachlorobenzene-1,4-dicarboxylato-κ2O1:O4)copper(II)] hemihydrate] Refinement

**Table:**

Refinement on *F*^2^	Primary atom site location: structure-invariant direct methods
Least-squares matrix: full	Secondary atom site location: difference Fourier map
*R*[*F*^2^ > 2σ(*F*^2^)] = 0.034	Hydrogen site location: mixed
*wR*(*F*^2^) = 0.109	H atoms treated by a mixture of independent and constrained refinement
*S* = 1.18	*w* = 1/[σ^2^(*F*_o_^2^) + (0.0575*P*)^2^ + 1.5531*P*] where *P* = (*F*_o_^2^ + 2*F*_c_^2^)/3
3684 reflections	(Δ/σ)_max_ = 0.002
242 parameters	Δρ_max_ = 0.49 e Å^−^^3^
5 restraints	Δρ_min_ = −0.66 e Å^−^^3^

### Poly[[aqua(µ2-pyrazine-κ2N:N')(µ2-2,3,5,6-tetrachlorobenzene-1,4-dicarboxylato-κ2O1:O4)copper(II)] hemihydrate] Special details

**Table:**

Geometry. All esds (except the esd in the dihedral angle between two l.s. planes) are estimated using the full covariance matrix. The cell esds are taken into account individually in the estimation of esds in distances, angles and torsion angles; correlations between esds in cell parameters are only used when they are defined by crystal symmetry. An approximate (isotropic) treatment of cell esds is used for estimating esds involving l.s. planes.

### Poly[[aqua(µ2-pyrazine-κ2N:N')(µ2-2,3,5,6-tetrachlorobenzene-1,4-dicarboxylato-κ2O1:O4)copper(II)] hemihydrate] Fractional atomic coordinates and isotropic or equivalent isotropic displacement parameters (Å^2^)

**Table:**

	*x*	*y*	*z*	*U*_iso_*/*U*_eq_	Occ. (<1)
Cu1	0.27764 (2)	0.62427 (4)	0.61712 (2)	0.01913 (11)	
Cl1	−0.09180 (7)	0.98567 (12)	0.58342 (4)	0.0420 (2)	
Cl2	−0.37150 (7)	0.92147 (13)	0.56485 (4)	0.0442 (2)	
Cl3	−0.34867 (7)	0.25931 (14)	0.69731 (4)	0.0493 (2)	
Cl4	−0.07035 (7)	0.31868 (14)	0.71717 (4)	0.0443 (2)	
O1	0.10094 (16)	0.6243 (3)	0.60367 (9)	0.0232 (4)	
O2	0.0908 (2)	0.7070 (5)	0.70164 (10)	0.0573 (8)	
O3	−0.49156 (19)	0.4414 (4)	0.56869 (10)	0.0404 (5)	
O4	−0.55433 (17)	0.6298 (3)	0.64610 (9)	0.0256 (4)	
O5	0.2948 (2)	0.6265 (4)	0.51195 (10)	0.0379 (5)	
O6	0.0506 (6)	0.5848 (12)	0.4800 (3)	0.0692 (19)	0.5
N1	0.27784 (19)	0.9216 (3)	0.62098 (10)	0.0217 (4)	
N2	0.27117 (19)	1.3279 (3)	0.62254 (10)	0.0222 (4)	
C1	−0.0938 (2)	0.6454 (4)	0.64777 (11)	0.0247 (5)	
C2	−0.1622 (2)	0.7822 (4)	0.61483 (12)	0.0259 (5)	
C3	−0.2862 (2)	0.7553 (4)	0.60668 (12)	0.0265 (5)	
C4	−0.3430 (2)	0.5911 (4)	0.63065 (12)	0.0231 (5)	
C5	−0.2765 (2)	0.4576 (4)	0.66547 (12)	0.0259 (5)	
C6	−0.1519 (2)	0.4851 (4)	0.67378 (11)	0.0252 (5)	
C7	0.0433 (2)	0.6632 (4)	0.65330 (12)	0.0258 (5)	
C8	−0.4746 (2)	0.5481 (4)	0.61336 (12)	0.0239 (5)	
C9	0.3304 (3)	1.0246 (4)	0.66616 (13)	0.0296 (6)	
H9	0.370398	0.957881	0.697589	0.036*	
C10	0.3264 (3)	1.2270 (4)	0.66709 (12)	0.0278 (6)	
H10	0.362814	1.294764	0.699390	0.033*	
C11	0.2177 (3)	1.2256 (4)	0.57790 (13)	0.0298 (6)	
H11	0.177402	1.292424	0.546606	0.036*	
C12	0.2209 (3)	1.0233 (4)	0.57707 (13)	0.0289 (6)	
H12	0.182535	0.955829	0.545269	0.035*	
H1	0.239 (3)	0.641 (6)	0.4894 (16)	0.044 (11)*	
H2	0.361 (3)	0.605 (8)	0.495 (2)	0.082 (18)*	
H3	0.035 (8)	0.592 (14)	0.5168 (12)	0.06 (3)*	0.5
H4	−0.003 (7)	0.515 (16)	0.465 (4)	0.09 (4)*	0.5

### Poly[[aqua(µ2-pyrazine-κ2N:N')(µ2-2,3,5,6-tetrachlorobenzene-1,4-dicarboxylato-κ2O1:O4)copper(II)] hemihydrate] Atomic displacement parameters (Å^2^)

**Table:**

	*U*^11^	*U*^22^	*U*^33^	*U*^12^	*U*^13^	*U*^23^
Cu1	0.01352 (17)	0.01482 (18)	0.02901 (18)	−0.00014 (10)	−0.00327 (11)	0.00023 (10)
Cl1	0.0272 (4)	0.0395 (4)	0.0592 (5)	−0.0092 (3)	−0.0032 (3)	0.0149 (3)
Cl2	0.0246 (4)	0.0445 (5)	0.0635 (5)	0.0030 (3)	−0.0061 (3)	0.0237 (4)
Cl3	0.0308 (4)	0.0530 (5)	0.0641 (5)	−0.0112 (3)	−0.0074 (4)	0.0311 (4)
Cl4	0.0297 (4)	0.0493 (5)	0.0539 (5)	0.0032 (3)	−0.0152 (3)	0.0175 (4)
O1	0.0121 (8)	0.0272 (10)	0.0302 (9)	0.0003 (6)	−0.0014 (7)	−0.0035 (7)
O2	0.0293 (12)	0.115 (2)	0.0270 (10)	−0.0183 (14)	−0.0058 (9)	−0.0104 (13)
O3	0.0232 (10)	0.0596 (15)	0.0383 (11)	−0.0023 (10)	−0.0007 (8)	−0.0149 (11)
O4	0.0152 (8)	0.0243 (10)	0.0371 (10)	0.0004 (7)	−0.0032 (7)	−0.0024 (7)
O5	0.0334 (12)	0.0501 (14)	0.0302 (11)	0.0059 (10)	0.0029 (9)	0.0006 (9)
O6	0.052 (3)	0.119 (6)	0.036 (3)	−0.019 (4)	−0.012 (2)	−0.021 (3)
N1	0.0211 (11)	0.0159 (10)	0.0282 (11)	−0.0002 (8)	−0.0051 (8)	0.0002 (8)
N2	0.0203 (10)	0.0165 (10)	0.0299 (11)	−0.0012 (8)	−0.0042 (8)	0.0014 (8)
C1	0.0153 (11)	0.0380 (15)	0.0209 (11)	−0.0011 (10)	−0.0011 (9)	−0.0031 (10)
C2	0.0171 (12)	0.0313 (14)	0.0293 (12)	−0.0034 (10)	0.0022 (9)	0.0011 (10)
C3	0.0195 (12)	0.0288 (14)	0.0312 (13)	0.0022 (10)	−0.0034 (10)	0.0021 (10)
C4	0.0117 (11)	0.0312 (14)	0.0264 (12)	0.0004 (9)	0.0008 (9)	−0.0009 (10)
C5	0.0170 (12)	0.0333 (14)	0.0274 (12)	−0.0018 (10)	0.0010 (9)	0.0037 (11)
C6	0.0148 (11)	0.0358 (15)	0.0249 (12)	0.0018 (10)	−0.0015 (9)	0.0018 (10)
C7	0.0143 (11)	0.0370 (15)	0.0261 (12)	−0.0030 (10)	−0.0017 (9)	0.0007 (11)
C8	0.0154 (11)	0.0272 (13)	0.0290 (12)	−0.0005 (10)	−0.0032 (9)	0.0032 (10)
C9	0.0359 (15)	0.0220 (13)	0.0308 (13)	−0.0010 (11)	−0.0131 (11)	0.0036 (10)
C10	0.0334 (14)	0.0216 (13)	0.0284 (12)	−0.0032 (11)	−0.0099 (11)	0.0005 (10)
C11	0.0350 (15)	0.0206 (13)	0.0335 (13)	−0.0004 (11)	−0.0173 (11)	0.0023 (10)
C12	0.0317 (14)	0.0201 (13)	0.0348 (14)	−0.0006 (11)	−0.0143 (11)	0.0010 (10)

### Poly[[aqua(µ2-pyrazine-κ2N:N')(µ2-2,3,5,6-tetrachlorobenzene-1,4-dicarboxylato-κ2O1:O4)copper(II)] hemihydrate] Geometric parameters (Å, º)

**Table:**

Cu1—O4^i^	1.9475 (19)	N1—C9	1.336 (3)
Cu1—O1	1.9603 (18)	N2—C11	1.330 (3)
Cu1—N2^ii^	2.015 (2)	N2—C10	1.332 (3)
Cu1—N1	2.019 (2)	C1—C6	1.383 (4)
Cu1—O5	2.301 (2)	C1—C2	1.392 (4)
Cl1—C2	1.725 (3)	C1—C7	1.514 (3)
Cl2—C3	1.724 (3)	C2—C3	1.384 (4)
Cl3—C5	1.710 (3)	C3—C4	1.381 (4)
Cl4—C6	1.722 (3)	C4—C5	1.387 (4)
O1—C7	1.283 (3)	C4—C8	1.520 (3)
O2—C7	1.211 (3)	C5—C6	1.392 (3)
O3—C8	1.227 (3)	C9—C10	1.374 (4)
O4—C8	1.260 (3)	C9—H9	0.9300
O5—H1	0.791 (19)	C10—H10	0.9300
O5—H2	0.82 (2)	C11—C12	1.373 (4)
O6—H3	0.82 (2)	C11—H11	0.9300
O6—H4	0.82 (2)	C12—H12	0.9300
N1—C12	1.334 (3)		
			
O4^i^—Cu1—O1	169.61 (8)	C4—C3—Cl2	118.9 (2)
O4^i^—Cu1—N2^ii^	91.93 (8)	C2—C3—Cl2	120.9 (2)
O1—Cu1—N2^ii^	88.49 (8)	C3—C4—C5	119.8 (2)
O4^i^—Cu1—N1	88.08 (8)	C3—C4—C8	119.4 (2)
O1—Cu1—N1	90.41 (8)	C5—C4—C8	120.5 (2)
N2^ii^—Cu1—N1	173.91 (9)	C4—C5—C6	119.9 (2)
O4^i^—Cu1—O5	104.00 (9)	C4—C5—Cl3	119.49 (19)
O1—Cu1—O5	86.33 (8)	C6—C5—Cl3	120.6 (2)
N2^ii^—Cu1—O5	93.91 (9)	C1—C6—C5	120.4 (2)
N1—Cu1—O5	91.99 (9)	C1—C6—Cl4	120.08 (19)
C7—O1—Cu1	111.38 (16)	C5—C6—Cl4	119.5 (2)
C8—O4—Cu1^iii^	117.82 (17)	O2—C7—O1	124.9 (2)
Cu1—O5—H1	124 (3)	O2—C7—C1	120.9 (2)
Cu1—O5—H2	121 (4)	O1—C7—C1	114.2 (2)
H1—O5—H2	115 (5)	O3—C8—O4	127.3 (2)
H3—O6—H4	105 (5)	O3—C8—C4	116.8 (2)
C12—N1—C9	117.3 (2)	O4—C8—C4	115.9 (2)
C12—N1—Cu1	119.10 (18)	N1—C9—C10	121.3 (2)
C9—N1—Cu1	123.59 (18)	N1—C9—H9	119.4
C11—N2—C10	117.6 (2)	C10—C9—H9	119.4
C11—N2—Cu1^iv^	119.57 (18)	N2—C10—C9	121.2 (2)
C10—N2—Cu1^iv^	122.65 (18)	N2—C10—H10	119.4
C6—C1—C2	119.2 (2)	C9—C10—H10	119.4
C6—C1—C7	119.3 (2)	N2—C11—C12	121.4 (2)
C2—C1—C7	121.5 (2)	N2—C11—H11	119.3
C3—C2—C1	120.4 (2)	C12—C11—H11	119.3
C3—C2—Cl1	119.8 (2)	N1—C12—C11	121.3 (2)
C1—C2—Cl1	119.8 (2)	N1—C12—H12	119.4
C4—C3—C2	120.2 (2)	C11—C12—H12	119.4
			
C6—C1—C2—C3	−1.9 (4)	Cl3—C5—C6—Cl4	−0.6 (3)
C7—C1—C2—C3	175.3 (2)	Cu1—O1—C7—O2	4.4 (4)
C6—C1—C2—Cl1	178.7 (2)	Cu1—O1—C7—C1	−174.14 (18)
C7—C1—C2—Cl1	−4.1 (4)	C6—C1—C7—O2	−73.1 (4)
C1—C2—C3—C4	−0.7 (4)	C2—C1—C7—O2	109.7 (4)
Cl1—C2—C3—C4	178.8 (2)	C6—C1—C7—O1	105.5 (3)
C1—C2—C3—Cl2	−178.9 (2)	C2—C1—C7—O1	−71.6 (4)
Cl1—C2—C3—Cl2	0.6 (3)	Cu1^iii^—O4—C8—O3	−10.5 (4)
C2—C3—C4—C5	3.1 (4)	Cu1^iii^—O4—C8—C4	168.05 (17)
Cl2—C3—C4—C5	−178.7 (2)	C3—C4—C8—O3	91.6 (3)
C2—C3—C4—C8	−170.9 (2)	C5—C4—C8—O3	−82.4 (3)
Cl2—C3—C4—C8	7.3 (4)	C3—C4—C8—O4	−87.1 (3)
C3—C4—C5—C6	−2.9 (4)	C5—C4—C8—O4	98.9 (3)
C8—C4—C5—C6	171.0 (2)	C12—N1—C9—C10	−0.4 (4)
C3—C4—C5—Cl3	177.6 (2)	Cu1—N1—C9—C10	−179.0 (2)
C8—C4—C5—Cl3	−8.4 (4)	C11—N2—C10—C9	1.5 (4)
C2—C1—C6—C5	2.0 (4)	Cu1^iv^—N2—C10—C9	−173.5 (2)
C7—C1—C6—C5	−175.2 (2)	N1—C9—C10—N2	−0.8 (5)
C2—C1—C6—Cl4	−177.6 (2)	C10—N2—C11—C12	−1.1 (4)
C7—C1—C6—Cl4	5.2 (3)	Cu1^iv^—N2—C11—C12	174.1 (2)
C4—C5—C6—C1	0.3 (4)	C9—N1—C12—C11	0.8 (4)
Cl3—C5—C6—C1	179.8 (2)	Cu1—N1—C12—C11	179.5 (2)
C4—C5—C6—Cl4	179.9 (2)	N2—C11—C12—N1	−0.1 (5)

Symmetry codes: (i) *x*+1, *y*, *z*; (ii) *x*, *y*−1, *z*; (iii) *x*−1, *y*, *z*; (iv) *x*, *y*+1, *z*.

### Poly[[aqua(µ2-pyrazine-κ2N:N')(µ2-2,3,5,6-tetrachlorobenzene-1,4-dicarboxylato-κ2O1:O4)copper(II)] hemihydrate] Hydrogen-bond geometry (Å, º)

**Table:**

*D*—H···*A*	*D*—H	H···*A*	*D*···*A*	*D*—H···*A*
O5—H1···O6	0.79 (2)	2.11 (3)	2.781 (7)	143 (4)
O5—H2···O3^v^	0.82 (2)	2.03 (3)	2.827 (3)	163 (5)
O6—H3···O1	0.82 (2)	2.04 (5)	2.763 (6)	147 (8)
O6—H4···O1^v^	0.82 (2)	2.07 (4)	2.843 (6)	156 (10)

Symmetry code: (v) −*x*, −*y*+1, −*z*+1.
